# Supplementary material for: HIVprotI: an integrated web based platform for prediction and design of HIV proteins inhibitors
Source: J Cheminform. 2018 Mar 9;10:12. doi: 10.1186/s13321-018-0266-y (PMC5845081; doi:10.1186/s13321-018-0266-y)
Supplement: Supplementary file 2 — Additional file 2. Source code of HIVProtI web server. [file 13321_2018_266_MOESM2_ESM.zip › HIVprotI_Source-code/hivprotI/weka-3-6-6/doc/weka/gui/visualize/PlotData2D.html]

PlotData2D


---


|  |  |  |
| --- | --- | --- |
| **Overview** \| **Package** \| **Class** \| **Tree** \| **Deprecated** \| **Index** \| **Help** \| **Weka's home** | |  |
| **PREV CLASS**   **NEXT CLASS** | **FRAMES**    **NO FRAMES**     **All Classes** |  |
| SUMMARY: NESTED \| FIELD \| CONSTR \| METHOD | DETAIL: FIELD \| CONSTR \| METHOD |  |


---


## weka.gui.visualize Class PlotData2D

```
java.lang.Object
  weka.gui.visualize.PlotData2D
```

---

``` public class PlotData2D extends java.lang.Object ```

This class is a container for plottable data. Instances form the
primary data. An optional array of classifier/clusterer predictions
(associated 1 for 1 with the instances) can also be provided.

**Version:**
:   $Revision: 7059 $

**Author:**
:   Mark Hall (mhall@cs.waikato.ac.nz)

---

| **Field Summary** | |
| --- | --- |
| `int` | `m_alwaysDisplayPointsOfThisSize`             If the shape size of a point equals this size then always plot it (i.e. |
| `java.awt.Color` | `m_customColour` |
| `boolean` | `m_displayAllPoints`             Display all points (ie. |
| `boolean` | `m_useCustomColour`             Custom colour for this plot |


**Constructor Summary**

`PlotData2D(Instances insts)`
  
          Construct a new PlotData2D using the supplied instances


| **Method Summary** | |
| --- | --- |
| `void` | `addInstanceNumberAttribute()`             Adds an instance number attribute to the plottable instances, |
| `int` | `getCindex()`             Get the currently set colouring index of the data |
| `Instances` | `getPlotInstances()`             Returns the instances for this plot |
| `java.lang.String` | `getPlotName()`             Get the name of this plot |
| `java.lang.String` | `getPlotNameHTML()`             Get the name of the plot for use in a tool tip text. |
| `int` | `getXindex()`             Get the currently set x index of the data |
| `int` | `getYindex()`             Get the currently set y index of the data |
| `void` | `setCindex(int c)`             Set the colouring index of the data |
| `void` | `setConnectPoints(boolean[] cp)`             Set whether consecutive points should be connected by lines |
| `void` | `setConnectPoints(FastVector cp)`             Set whether consecutive points should be connected by lines |
| `void` | `setCustomColour(java.awt.Color c)`             Set a custom colour to use for this plot. |
| `void` | `setPlotName(java.lang.String name)`             Set the name of this plot |
| `void` | `setPlotNameHTML(java.lang.String name)`             Set the plot name for use in a tool tip text. |
| `void` | `setShapeSize(FastVector ss)`             Set the shape sizes for the plot data |
| `void` | `setShapeSize(int[] ss)`             Set the shape sizes for the plot data |
| `void` | `setShapeType(FastVector st)`             Set the shape type for the plot data |
| `void` | `setShapeType(int[] st)`             Set the shape type for the plot data |
| `void` | `setXindex(int x)`             Set the x index of the data. |
| `void` | `setYindex(int y)`             Set the y index of the data |

**Methods inherited from class java.lang.Object**

`equals, getClass, hashCode, notify, notifyAll, toString, wait, wait, wait`

**Field Detail**

### m\_useCustomColour

```
public boolean m_useCustomColour
```

:   Custom colour for this plot

---


### m\_customColour

```
public java.awt.Color m_customColour
```

---


### m\_displayAllPoints

```
public boolean m_displayAllPoints
```

:   Display all points (ie. those that map to the same display coords)

---


### m\_alwaysDisplayPointsOfThisSize

```
public int m_alwaysDisplayPointsOfThisSize
```

:   If the shape size of a point equals this size then always plot
    it (i.e. even if it is obscured by other points)


**Constructor Detail**

### PlotData2D

```
public PlotData2D(Instances insts)
```

:   Construct a new PlotData2D using the supplied instances

    **Parameters:**: `insts` - the instances to use.


**Method Detail**

### addInstanceNumberAttribute

```
public void addInstanceNumberAttribute()
```

:   Adds an instance number attribute to the plottable instances,

---


### getPlotInstances

```
public Instances getPlotInstances()
```

:   Returns the instances for this plot

    :   **Returns:**: the instances for this plot

---


### setPlotName

```
public void setPlotName(java.lang.String name)
```

:   Set the name of this plot

    :   **Parameters:**: `name` - the name for this plot

---


### getPlotName

```
public java.lang.String getPlotName()
```

:   Get the name of this plot

    :   **Returns:**: the name of this plot

---


### setPlotNameHTML

```
public void setPlotNameHTML(java.lang.String name)
```

:   Set the plot name for use in a tool tip text.

    :   **Parameters:**: `name` - the name of the plot for potential use in a tool tip text (may use html).

---


### getPlotNameHTML

```
public java.lang.String getPlotNameHTML()
```

:   Get the name of the plot for use in a tool tip text.
    Defaults to the standard plot name if it hasn't been set.

    :   **Returns:**: the name of this plot (possibly in html) for use in a tool tip text.

---


### setShapeType

```
public void setShapeType(int[] st)
                  throws java.lang.Exception
```

:   Set the shape type for the plot data

    :   **Parameters:**: `st` - an array of integers corresponding to shape types (see constants defined in Plot2D) **Throws:**: `java.lang.Exception`

---


### setShapeType

```
public void setShapeType(FastVector st)
                  throws java.lang.Exception
```

:   Set the shape type for the plot data

    :   **Parameters:**: `st` - a FastVector of integers corresponding to shape types (see constants defined in Plot2D) **Throws:**: `java.lang.Exception`

---


### setShapeSize

```
public void setShapeSize(int[] ss)
                  throws java.lang.Exception
```

:   Set the shape sizes for the plot data

    :   **Parameters:**: `ss` - an array of integers specifying the size of data points **Throws:**: `java.lang.Exception`

---


### setShapeSize

```
public void setShapeSize(FastVector ss)
                  throws java.lang.Exception
```

:   Set the shape sizes for the plot data

    :   **Parameters:**: `ss` - a FastVector of integers specifying the size of data points **Throws:**: `java.lang.Exception`

---


### setConnectPoints

```
public void setConnectPoints(boolean[] cp)
                      throws java.lang.Exception
```

:   Set whether consecutive points should be connected by lines

    :   **Parameters:**: `cp` - an array of boolean specifying which points should be connected to their preceeding neighbour. **Throws:**: `java.lang.Exception`

---


### setConnectPoints

```
public void setConnectPoints(FastVector cp)
                      throws java.lang.Exception
```

:   Set whether consecutive points should be connected by lines

    :   **Parameters:**: `cp` - a FastVector of boolean specifying which points should be connected to their preceeding neighbour. **Throws:**: `java.lang.Exception`

---


### setCustomColour

```
public void setCustomColour(java.awt.Color c)
```

:   Set a custom colour to use for this plot. This overides any
    data index to use for colouring. If null, then will revert back
    to the default (no custom colouring).

    :   **Parameters:**: `c` - a custom colour to use for this plot or null (default---no colouring).

---


### setXindex

```
public void setXindex(int x)
```

:   Set the x index of the data.

    :   **Parameters:**: `x` - the x index

---


### setYindex

```
public void setYindex(int y)
```

:   Set the y index of the data

    :   **Parameters:**: `y` - the y index

---


### setCindex

```
public void setCindex(int c)
```

:   Set the colouring index of the data

    :   **Parameters:**: `c` - the colouring index

---


### getXindex

```
public int getXindex()
```

:   Get the currently set x index of the data

    :   **Returns:**: the current x index

---


### getYindex

```
public int getYindex()
```

:   Get the currently set y index of the data

    :   **Returns:**: the current y index

---


### getCindex

```
public int getCindex()
```

:   Get the currently set colouring index of the data

    :   **Returns:**: the current colouring index


---


|  |  |  |
| --- | --- | --- |
| **Overview** \| **Package** \| **Class** \| **Tree** \| **Deprecated** \| **Index** \| **Help** \| **Weka's home** | |  |
| **PREV CLASS**   **NEXT CLASS** | **FRAMES**    **NO FRAMES**     **All Classes** |  |
| SUMMARY: NESTED \| FIELD \| CONSTR \| METHOD | DETAIL: FIELD \| CONSTR \| METHOD |  |


---
